# Electrical Osmosis

**Published:** 1896-06

**Authors:** 


### ELECTRICAL OSMOSIS.

This new form of treating sensitive dentine is justly claiming a large share of attention. The earlier efforts in this direction and the conclusions reached have been confirmed by subsequent experiments.

Dr. Jack gives us a very practical paper in this number, and Dr. Brown, of Montreal, has supplemented his able paper of April with additional facts.

In an interesting clinic given to a few deeply-interested observers by Dr. Jack, we were impressed by two facts,—first, the possibility of carrying the agent used—in this case cocaine—through apparently dense dentine. The case operated upon was that of an inferior bicuspid, abraded to an even surface, but exquisitely sensitive, so much so that the touch of the finger produced a painful shock. The current was turned on carrying a twelve-per-cent. solution of cocaine, and in a short time all sensation disappeared. The excavator demonstrated, however, that this obtunding was superficial, and a second application was rendered necessary, but with entire success.

The point of interest here, outside of the loss of sensation, was the fact that an abraded tooth means an almost certain increased calcification of the tubuli and a supposed increased resistance or, in any event, a greater obstacle to osmotic action, but in this instance the result was entirely satisfactory and quickly produced.

The second case was a large superficial cavity on the buccal surface of an inferior molar. This required twenty minutes, but a perfect obtunding was the result. The singular fact was developed that these teeth, insensible to the excavator, quickly responded to thermal change by the use of cold water.

The time has not yet arrived when the question can be answered, what effect will this treatment have on the pulp? The danger, if danger exists, will be in that direction. Theoretically the paralyzing effect of the cocaine should, in some cases, result in an eventual devitalization of the pulp. As the action seems to be superficial, this could only occur in cavities with a thin layer of dentine over the central organ.

The experience that some have had in the production of great pain, seems to have been the result of inadequate instruments or methods of manipulation, for with the apparatus described by Dr. Jack it would seem impossible that, with ordinary care, this could occur.

Another complaint made is the time taken out of an operator's hour, from twelve to twenty minutes. This is, apparently, a serious objection, and yet it is only apparent, for the time lost struggling with a nervous patient, to say nothing of the unending strain upon the dentist, more than counterbalances the delay, for the time lost is fully made up by subsequent rapidity of execution.

There is another difficulty yet to be considered, and it is by no means a slight one, the danger of filling over an exposed and temporarily obtunded pulp. This in careless hands will certainly be of frequent occurrence. Notwithstanding the many doubts surrounding this new method, the outcome will be awaited with increasing interest.

